# Divanillyl sulfone suppresses NLRP3 inflammasome activation via inducing mitophagy to ameliorate chronic neuropathic pain in mice

**DOI:** 10.1186/s12974-021-02178-z

**Published:** 2021-06-24

**Authors:** Shuai Shao, Cheng-Bo Xu, Cheng-Juan Chen, Gao-Na Shi, Qing-Lan Guo, Yu Zhou, Ya-Zi Wei, Lei Wu, Jian-Gong Shi, Tian-Tai Zhang

**Affiliations:** State Key Laboratory of Bioactive Substances and Functions of Natural Medicines, Institute of Materia Medica, Chinese Academy of Medical Sciences & Peking Union Medical College, Beijing, 100050 China

**Keywords:** Chronic neuropathic pain, Microglia, Mitophagy, NLRP3 inflammasome, Divanillyl sulfone

## Abstract

**Background:**

Chronic neuropathic pain is a frequent sequel to peripheral nerve injury and maladaptive nervous system function. Divanillyl sulfone (DS), a novel structural derivative of 4,4′-dihydroxydibenzyl sulfoxide from a traditional Chinese medicine *Gastrodia elata* with anti-nociceptive effects, significantly alleviated neuropathic pain following intrathecal injection. Here, we aimed to investigate the underlying mechanisms of DS against neuropathic pain.

**Methods:**

A chronic constrictive injury (CCI) mouse model of neuropathic pain induced by sciatic nerve ligation was performed to evaluate the effect of DS by measuring the limb withdrawal using Von Frey filament test. Immunofluorescence staining was used to assess the cell localizations and expressions of Iba-1, ASC, NLRP3, and ROS, the formation of autolysosome. The levels of NLRP3-related proteins (caspase-1, NLRP3, and IL-1β), mitophagy-related proteins (LC3, Beclin-1, and p62), and apoptosis-related proteins (Bcl-XL and Bax) were detected by Western blotting. The apoptosis of BV-2 cell and caspase activity were evaluated by flow cytometry.

**Results:**

DS significantly alleviated the neuropathic pain by increasing the mechanical withdrawal threshold and inhibiting the activation of NLRP3 in CCI-induced model mice. Our findings indicated that DS promoted the mitophagy by increasing the LC3II and Beclin 1 and decreasing the levels of p62 protein in BV-2 cell. This is accompanied by the inhibition of NLRP3 activation, which was shown as inhibited the expression of NLRP3 in lysates as well as the secretion of mature caspase-1 p10 and IL-1β p17 in supernatants in cultured BV-2 microglia. In addition, DS could promote mitophagy-induced improvement of dysfunctional mitochondria by clearing intracellular ROS and restoring mitochondrial membrane potential.

**Conclusion:**

Together, our findings demonstrated that DS ameliorate chronic neuropathic pain in mice by suppressing NLRP3 inflammasome activation induced by mitophagy in microglia. DS may be a promising therapeutic agent for chronic neuropathic pain.

**Supplementary Information:**

The online version contains supplementary material available at 10.1186/s12974-021-02178-z.

## Background

Neuralgia is a type of chronic pain that manifests as spontaneous pain, hyperalgesia, and allodynia [1, 2]. It is caused by injury or disease in the somatosensory nervous system that includes the central nerves, spinal cord, posterior root of the spinal cord, and peripheral nerves. Pre-clinical studies show that the basis of neuropathic pain is the plasticity of nerve cells [3, 4]. In addition, some non-neuronal cells of the central nervous system (CNS), especially microglia, have also been implicated in triggering neuropathic pain [5–8].

Microglia is a kind of primary innate immune cells in the CNS, and is activated by various pathological stimuli. The activated microglia interacts with the astroglia or neurons to induce neuroinflammation and facilitate transmission of pain signals [9–11]. Chronic neuropathic pain is characterized by infiltration of immune cells into the dorsal root ganglia (DRG), and the activation of microglia in spinal cord and brain, eventually leading to a neuroinflammatory response [12]. Pro-inflammatory factor of interleukin-1β (IL-1β) plays an important role in the microglial inflammatory signaling mediated neuropathic pain [13]. Multiple mechanisms participate in the central neuronal excitation mediated by microglial inflammation in peripheral nerve injury (PNI)-induced neuropathic pain. In microglia, the activation of toll-like receptor 2 (TLR2) and/or TLR4 can promote nuclear factor-κB (NF-κB) signal to induce IL-1β transcription [14], triggering receptor expressed on myeloid cells 2 (TREM2) and via transcription factors interferon regulatory factor 1/5/8 (IRF1/5/8) [15]. In addition, P2X purinoceptor 7 (P2X7), P2Y purinoceptor 12 (P2Y12), and CX3C-chemokine receptor 1 (CX3CR1) on microglial cells also lead to IL-1β secretion via p38 MAPKs as well [16]. The important thing is that nucleotide-binding oligomerization domain (NOD), leucine-rich repeat, and pyrin domains-containing protein (NLRP)-type inflammasomes promote pro-IL-1β processing and mature IL-1β secretion. Of NLR family, NLR family pyrin domain-containing 3 (NLRP3) is the most extensively studied and well-characterized inflammasome sensor molecule. Meanwhile, NLRP3 inflammasome can also be activated by TLRs receptor and P2X7. IL-1β acts as mediators between microglia and neurons and assumes roles as neuromodulator when it acts on spinal dorsal horn (SDH) neurons to increase the strength of synaptic connectivity and excitatory synaptic transmission in the process of neuropathic pain [17, 18]. Thereby, the NLRP3 inflammasome is the most recognized contributor and plays an irreplaceable role to the transmission of pain signals [19, 20].

NLRP3 inflammasome is a multi-protein complex consisting of pattern recognition receptors (PRRs) that recognize pathogen-associated molecular patterns (PAMPs) and danger-associated molecular patterns (DAMPs) during the course of natural immune response, which then activates caspase-1 and promotes the maturation and secretion of pro-inflammatory cytokines like IL-1β and interleukin-18 (IL-18) [16, 21]. The NLRP3 inflammasome/IL-1 pathway shows outstanding advantages in the process of neuralgia signal transduction. The IL-1β secreted from the microglia binds to the IL-1 receptor (IL-1R) expressed on SDH neurons, which promotes *N*-methyl-d-aspartate receptor (NMDAR) phosphorylation and reverses γ-aminobutyrate (GABA) and/or glycine-mediated synaptic inhibition, thereby enhancing transmission of excitatory signals [11, 22, 23]. IL-1R has two isoforms of IL-1R1 and IL-1R2. In contrast to IL-1R1, IL-1R2 is a decoy receptor and negative regulator of the IL-1 pathway, just like IL-1R antagonist (IL-1Ra). IL-1Ra, one of the members of IL-1 family acting as a natural IL-1 inhibitor, could bind to both IL-1R1 and IL-1R2 to prevent uncontrolled activation of IL-1 receptors by competing IL-1β or IL-1α for binding [24]. Although IL-1Ra or IL-1β neutralizing antibodies are promising analgesics [25–27], complete blockade of IL-1β/IL-1R1 will have an immunosuppressive effect that may increase the risk of infections [17]. Therefore, selective inhibition of IL-1β secretion by activated NLRP3 inflammasome is a better strategy to alleviate neuropathic pain, since the non-canonical pathway of NLRP3 activation and other inflammasomes can still ensure IL-1β secretion for normal physiological functions [28]. To this end, direct inhibitors of NLRP3 inflammasome or activation pathway blockers are increasingly gaining attention for their potential analgesic effects.

The NLRP3 inflammasome can be activated by K^+^ efflux, Reactive oxygen species (ROS) releasing, and lysosomal disruption [29, 30]. Mitochondrial damage is the primary trigger of excessive ROS production and lysosomal dysfunction, which might induce obvious activation of NLRP3 inflammasome [31]. However, mitophagy may block the activation of NLRP3 inflammasome and subsequent of pain signal transmission [32, 33]. Autophagy is an adaptive response of eukaryotic cells to stressful stimuli wherein damaged cellular components are recycled via formation of autophagosomes. Selective elimination of damaged mitochondria through mycophagy in the microglia is crucial for neuron survival and transmission of pain signals [34]. Furthermore, mitophagy can inhibit NLRP3 inflammasome activation and reduce the secretion of IL-1β, most likely by preventing mitochondrial ROS production and reversing mitochondrial membrane depolarization [35]. Therefore, NLRP3 inflammasome inhibitors associated with mitophagy induction is worth investigating as analgesics against neuropathic pain.

*Gastrodia elata* Blume is a traditional Chinese medicine used for the treatment of headache, migraine, and other neuropathic pain, from which gastrodin was isolated as the main active constituents. Divanillyl sulfone (DS) is a derivative of 4,4′-dihydroxydibenzyl sulfoxide, which also was reported as the active constitute of *Gastrodia elata* Blume [36]. In the preliminary evaluation, DS showed potential pharmacological activity against neuropathic pain. In this study, we established somatic and neuropathic pain models to evaluate the anti-nociceptive effects of DS and explored the underlying mechanisms involving NLRP3 inflammasome activation in the microglia.

## Materials and methods

### Drugs and reagents

DS was synthesized and gastrodin was extracted and purified by the Institute of Materia Medica, Chinese Academy of Medical Sciences and Peking Union Medical College, and the purity was validated as > 95% using high-performance liquid chromatography. Stock solution of both reagents were prepared in dimethyl sulfoxide (DMSO) and subsequently diluted in sterile 0.9% saline. The cell culture reagents were purchased from Invitrogen Corporation (Thermo Fisher Scientific, Carlsbad, CA, USA). Antibodies for Western blotting and immunofluorescence were purchased from Abcam (Cambridge, UK) and Cell Signaling Technology (Beverly, MA, USA). Alexa 546-conjugated goat anti-rabbit and Alexa 488-conjugated goat anti-mouse secondary antibodies were from Life Technologies (Thermo Fisher Scientific, Carlsbad, CA, USA). Goat serum, Mito-Tracker Green, Lyso-Tracker Red, and Rh123 were purchased from Beyotime Biotechnology (Shanghai, China). Nigericin, 3-methyladenine (3-MA), and MCC950 were purchased from MedChemExpress (Monmouth Junction, NJ); lipopolysaccharide (LPS) and tritonX-100 from Sigma-Aldrich (St. Louis, MO); and recombinant IL-1Ra from PeproTech (Rocky Hill, NJ, USA). CellROX® Deep Red Reagent was purchased from Invitrogen (Carlsbad, CA, USA), annexin-V/7aad staining kit from BD Biosciences (Franklin Lakes, NJ, USA), and FAM-FLICA® caspase assay kit from Immunochemistry Technologies (Bloomington, MN, USA).

### Experimental animals

Male ICR mice and C57BL/6 mice (weighing 18–20 g) were obtained from Beijing Huafukang Experimental Animal Institute (Beijing, China). The mice were housed 5–6 per cage at room temperature (22 ± 2 °C) in specific pathogen-free conditions under a 12/12-h reversed light-dark cycle, with food and water provided ad libitum. The mice were acclimatized for 3–4 days prior to the experiments, and randomly divided into the different groups. All procedures were approved by the Experimental Animal Care and Use Committee of the Institute of Materia Medica, Chinese Academy of Medical Sciences & Peking Union Medical College.

### Establishment of somatic pain model and behavioral assessment

Acetic acid-induced somatic pain model was established to preliminarily evaluate the analgesic effect of DS. The ICR mice were given a single intraperitoneal injection of DS (1, 3, or 10 mg/kg), normal saline (10 mL/kg), or gastrodin (positive control; 80 mg/kg), followed by 1% acetic acid (10 mL/kg, i.p.) 30 min later. The numbers of writhing and stretching in mice were counted over a period of 15 min after acetic acid injection.

### Establishment of neuropathic pain model and behavioral analysis

Chronic neuropathic pain following peripheral nerve injury was simulated via chronic constrictive injury (CCI) of the unilateral sciatic nerve. Briefly, the C57BL/6 mice were anesthetized with isoflurane, and randomly divided into the sham-operated, untreated CCI model, DS-treated (1, 3, and 10 mg/kg) and gastrodin (80 mg/kg) groups (*n* = 8 mice per group). The left sciatic nerve trunk was exposed by blunt dissection at mid-thigh level, and 4 ligatures (4-0 chromic catgut) were tied loosely around the nerve with 1 mm spacing. In the sham-operated mice, the sciatic nerve was only exposed but not ligated. On the ninth day after surgery, the mice were given a single intrathecal injection of the suitable dose of DS or saline. Briefly, the mice were anesthetized with isoflurane (4% for induction and 1% for maintenance), and a 100 μL micro-injector was inserted from the intervertebral space between the L5 and L6 discs into the spinal subarachnoid space. After confirming proper intrathecal injection via tail flicking, 100 μL normal saline or drug was microinjected followed by a 100 μL normal saline flush.

The sensitivity of mechanical nociception was measured by the Von Frey withdrawal test (Von Frey filaments, IITC Life Science Inc, California, USA) after 30 min, 1 h, and 2 h of intrathecal injection over a period of 4 days. The animals were acclimatized in boxes set on an elevated metal mesh floor for at least 30 min. Pressure values were pressed vertically on the sole of the hind paws with increasing force till the animal withdraw the hind limb. The procedure was repeated 3 times for an average data, and the value of paw withdrawal threshold was recorded. All behavioral analyses were performed by an investigator blinded to the experimental grouping.

The half-effective dose (ED_50_) of DS was determined by Von Frey withdrawal test on CCI-induced neuropathic pain model mice. Model mice were treated with a serious dose of DS (1, 3, 10, 30, 100 mg/mg) to test the paw withdrawal threshold at 0.5 h, 1 h, and 2 h after administration. The values of peak time of paw withdrawal threshold were selected to calculate the ED_50_ of DS on CCI-induced model mice.

### BV-2 cells culture and treatment

BV-2 cells were cultured in Dulbecco’s modified eagle’s medium (DMEM) supplemented with 10% fetal bovine serum (FBS) at 37 °C under 5% CO_2_ and 95% humidity. The cells were pre-stimulated with LPS (100 ng/mL) for 3.5 h, and then with 5 μM nigericin for 30 min, 6 h, 12 h, and 24 h as appropriate. In addition, the cells were treated with varying concentrations of DS (0.3, 3, and 30 μM) or 3-MA (5 mM) for 1 h and 30 min respectively prior to nigericin.

### Extraction and separation of cell supernatant protein

Cell supernatant protein was extracted by the standard methanol/chloroform method. The cell supernatants were layered with 1/4 volume of chloroform, and the same volume of methanol was added. After evenly mixing the solutions on a vortex shaker, the mixture was centrifuged at 12,000 rpm for 5 min at room temperature. The upper aqueous phase (methanol) was carefully removed, and the intermediate protein layer was aspirated and extracted once with methanol as described above.

### Western blotting analysis

Proteins were quantified by BCA Protein Assay Kit (Thermo Fisher Scientific, Carlsbad, CA, USA). Equal amount of proteins per sample were separated by 8% and 15% sodium dodecyl sulfate-polyacrylamide gel electrophoresis (SDS-PAGE), and the bands were transferred to a polyvinylidene fluoride (PVDF) membrane (Millipore Corp., Bedford, MA, USA). After blocking with 5% bovine serum albumin (BSA) in tris-based saline-Tween 20 (TBST) at room temperature for 1 h, the blots were incubated overnight with primary antibodies against IBA-1, NLRP3, pro-caspase-1, caspase-1, pro-IL-1β, IL-1β, LC3II/I, Beclin 1, protein 62 (p62), and β-tubulin (all diluted 1:1000) at 4 °C. Following incubation with horseradish peroxidase (HRP)-conjugated goat anti-rabbit and goat anti-mouse secondary antibodies, the blots were developed using enhanced chemiluminescence reagents (Perkinelmer, USA). The positive bands were visualized with Tanon 2000 Imaging System (Beijing, China) and their intensities were quantified using ImageJ Software (NIH, USA).

### Enzyme-linked immunosorbent assay

The level of IL-1β in the supernatant from cultured BV-2 cells was measured by enzyme-linked immunosorbent assay (ELISA) kit (Cat. No. 432606, BioLegend) according to the manufacturer’s instructions. The optical density (OD) at 450 nm was obtained by an ELISA plate reader (Synergy H1, BioTek, USA).

### Immunofluorescence

BV-2 cells were fixed with 4% paraformaldehyde for 30 min at room temperature, and permeabilized with 0.2% triton X-100 in phosphate buffered saline (PBS) containing 10% goat serum for 30 min. The cells were then incubated overnight with anti-NLRP3 (1:200) and anti-ASC (1:200) primary antibodies at 4 °C, washed with PBS, and probed with donkey anti-goat Alexa Fluor 488- and goat anti-rabbit Alexa Fluor 546-conjugated secondary antibodies (1:200) for 1 h at 37 °C. The nuclei were counterstained with DAPI (1 μg/mL), washed thrice with PBS, and observed under the Leica TCS SP8 confocal microscope (Leica Microsystems GmbH, Mannheim, Germany). Spinal cord tissues from the CCI mice were also stained with anti-NLRP3 and anti-IBA-1 antibodies as described above.

### Mitochondrial and lysosomal imaging

BV-2 cells were seeded in laser confocal plate (Nest, Jiangsu, China), and incubated with 75 nM Lyso-Tracker Red and 150 nM Mito-Tracker Green at 37 °C for 1 h. The nuclei were counterstained with DAPI (1 μg/mL), washed thrice with PBS, and observed under the Leica TCS SP8 confocal microscope.

### Mitochondrial membrane potential and reactive oxygen species measurement

Mitochondrial membrane potential (MMP) was measured using the cationic fluorescent probe Rh123 which rapidly translocated from the mitochondrial membrane to the matrix following membrane depolarization. The reactive oxygen species (ROS) levels were detected using CellROX Deep Red, which emits a strong fluorescence signal under oxidizing conditions. The suitably treated cells were incubated with 10 μM Rh123 or 5 μM CellROX Deep Red at room temperature for 30 min, and 1 μg/mL DAPI was added 10 min before the end of staining. After washing with PBS, the cells were observed under the Leica TCS SP8 confocal microscope to determine the fluorescence intensities of the respective probes.

### Flow cytometry

BV-2 cells were cultured and prepared to detect by flow cytometric staining with propidium iodide (PI)-PerCP and caspase-1-FITC (FAM-YVAD-FMK, Immunochemistry Technologies) for NLRP3 inflammasome activation; PI-PerCP and caspase-3/7-FITC (FAM-DEVD-FMK, Immunochemistry Technologies), and 7aad-PerCP and Annexin-V-APC for apoptosis (BD Biosciences). Briefly, the cultured cells at 37 °C were stimulated by LPS (100 ng/mL) for 3.5 h, incubation with or without 3-MA (5 mM) for another 30 min, and then incubation with DS (0.3, 3, and 30 μM for apoptosis measurement; 30 μM for NLRP3 inflammasome activation measurement) for 1 h. After stimulating with nigericin (5 μM), the harvested cells were washed twice with ice-cold PBS, and stained with 7aad or PI for 5 min at room temperature, Annexin-V for 15 min at room temperature, or FAM-YVAD-FMK (caspase-1) and FAM-DEVD-FMK (caspase-3/7) for 1 h at 37 °C, respectively. The samples were acquired in a FACS BD verse cytometer (BD Biosciences), and data were analyzed using FlowJo software (Version 10.0; Three Star).

### Statistical analysis

Data were presented as the mean ± SEM of at least 3 independent experiments. The paw withdrawal threshold (PWT (g)) was measured with Von Frey test and compared by two-factor analysis of variance (ANOVA) followed by Tukey’s post hoc test. The percentage of analgesia was calculated using the formula: (post-DS threshold in ipsilateral paw − baseline threshold in ipsilateral paw) / (baseline threshold in contralateral paw − baseline threshold in ipsilateral paw) × 100. Analysis of ED_50_ was performed with GraphPad best projected by the non-linear least-squares method in which data is normalized according to the maximal possible effect. The other parameters were analyzed using one-way ANOVA followed by an appropriate post hoc test, and *P <* 0.05 was considered statistically significant. Statistical analysis was performed using GraphPad Prism 8.01 software (GraphPad Software Inc., CA, USA).

## Results

### DS attenuated somatic pain in vivo

**Fig. 1:**
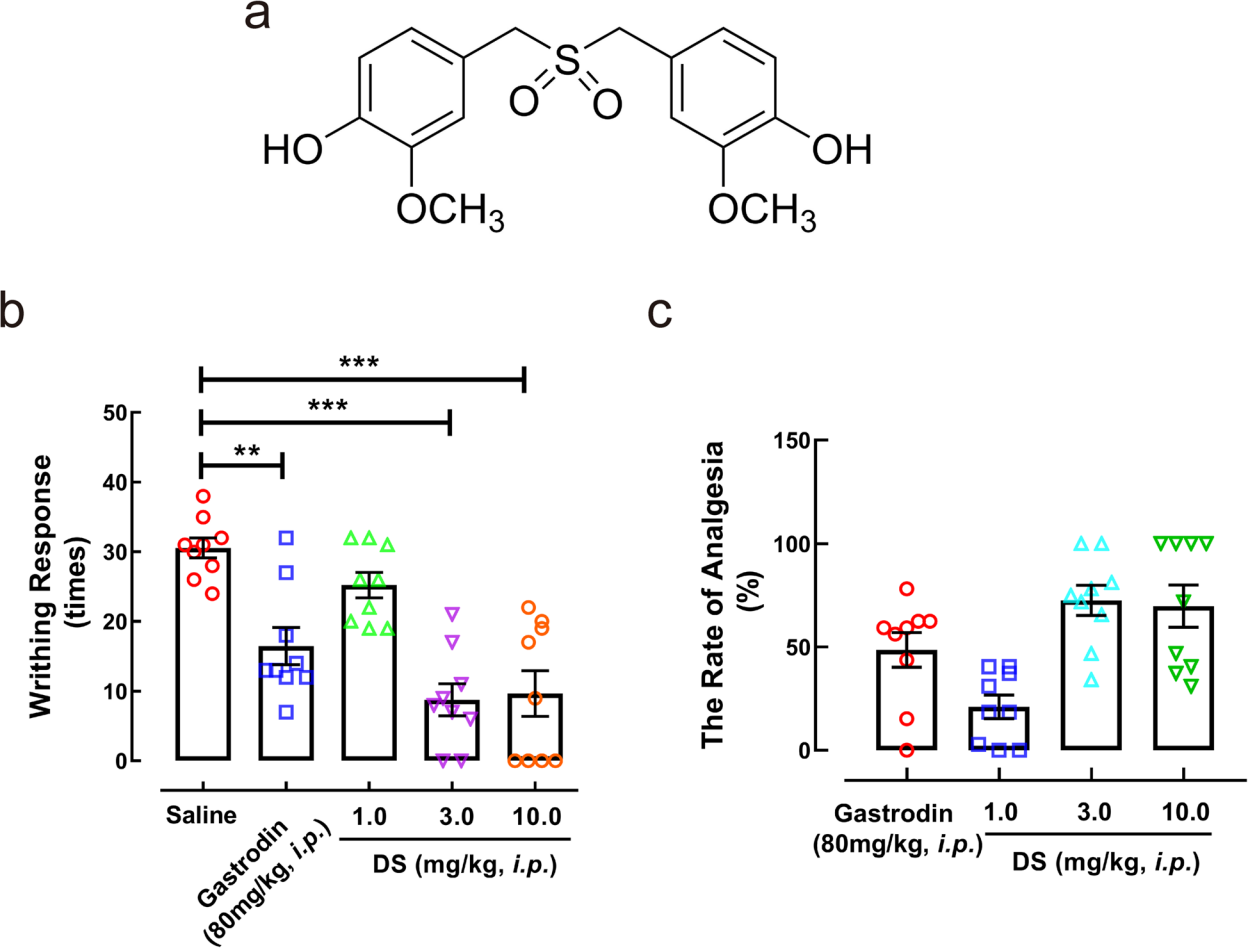
DS attenuated pain hypersensitivity of somatic pain in vivo. **a** The chemical structure of DS. DS significantly decreased the writhing times (**b**) and increased the rate of analgesia (**c**) in acetic acid-induced mice. Data are expressed as mean ± SEM (*n* = 9 mice in each group). Statistical significance was determined by one-way ANOVA followed by Tukey’s post hoc analysis where ^**^P < 0.01, ^***^P < 0.001 vs. saline group

DS, 4,4′-[Sulfonyl bis(methylene)] bis(2-methoxyphenol) (Fig. 1a), is obtained by acetic acid/hydrogen peroxide oxidation of 4,4′-[sulfinyl bis(methylene)] diphenol. We established a mouse model of somatic pain by injecting ICR mice with low concentration of acetic acid (1%), and found that intraperitoneal administration of DS significantly reduced the number of writhing behavior compared to the placebo in a dose-dependent manner. In addition, the analgesic effect of 1, 3, and 10 mg/kg DS were respectively 21.00%, 72.48%, and 74.53% compared to 48.48% observed with 80 mg/kg gastrodin (Fig. 1b, c).

### DS alleviated CCI-induced neuropathic pain in vivo

**Fig. 2:**
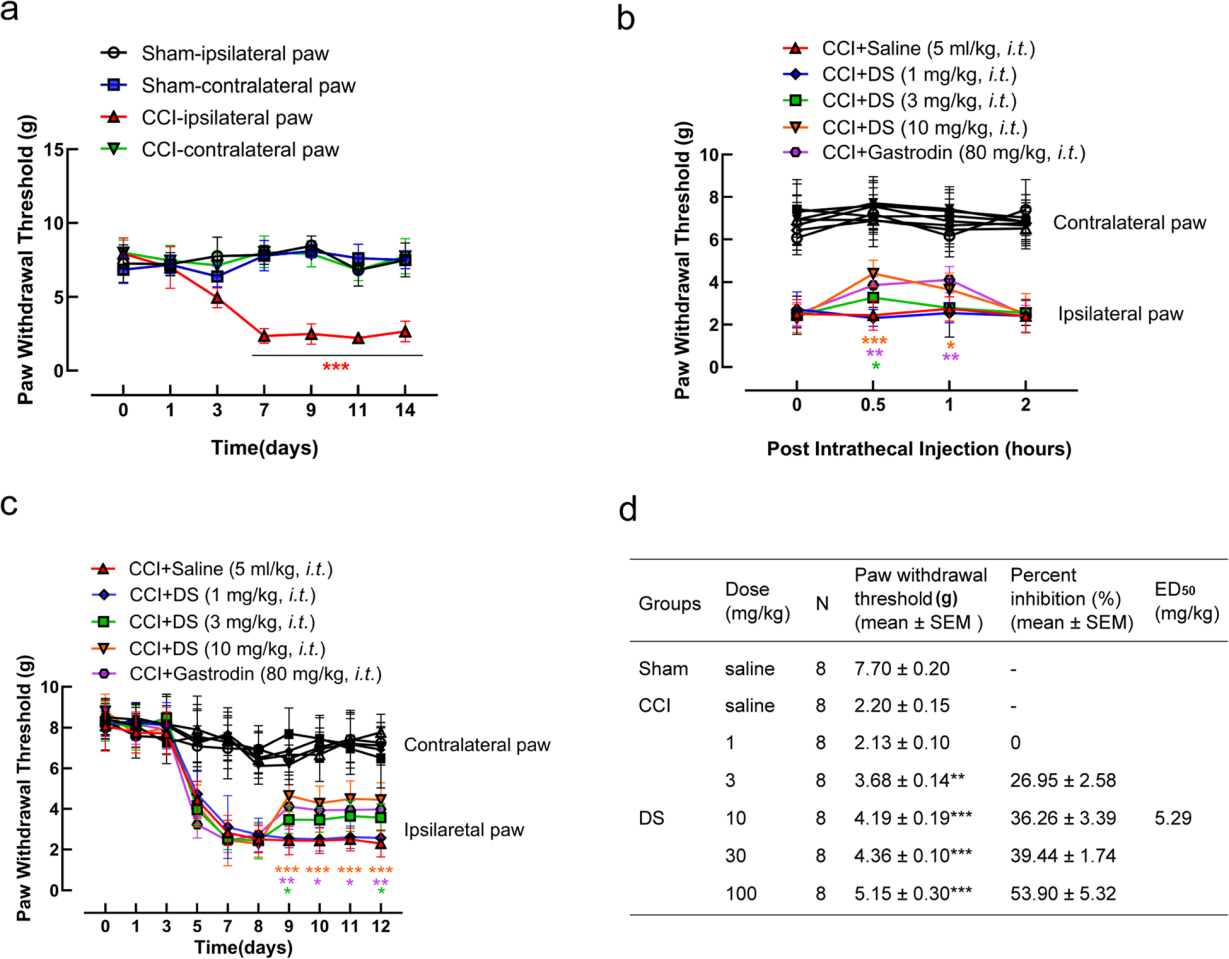
DS treatment improved sciatic nerve ligation-induced chronic neuropathic pain. Neuropathic pain mice were induced by ligation of sciatic nerve, the mechanical withdrawal threshold within 14 days after surgery (**a**) and intrathecal injection of vehicle (0.9% normal saline) or DS (1, 3, 10 mg/kg) 9 days after surgery (**b**) were measured by Von Frey Monofilament in both contralateral and ipsilateral paws. **c** The analgesic effects of both drugs over four consecutive days post-injection. **d** Dose-response analysis of DS on paw withdrawal threshold 0.5 h after administration, and ED_50_ was calculated with GraphPad Prism 8 with normalized to maximal possible effect. Data are expressed as means ± SEM (*n* = 8 mice in each group). Statistical significance was determined by two-way ANOVA followed by Tukey’s post hoc analysis where ^*^P < 0.05, ^**^P < 0.01, ^***^P < 0.001 vs. CCI + Saline group, ^***^P < 0.001 vs. Sham-ipsilateral paw group

A mouse model of chronic neuropathic pain was established by sciatic nerve ligation, and the effect of DS on mechanical allodynia was evaluated in terms of paw withdrawal. Sciatic nerve ligation decreased the paw withdrawal threshold of the ipsilateral but not the contralateral paw, and the threshold reached its lowest point on the seventh day post-surgery and maintained at this value for the rest of testing period, indicating that the neuropathic pain model was successfully induced (Fig. 2a). However, intrathecal injection of DS increased paw withdrawal threshold in the ipsilateral paw in a dose-dependent manner compared to the placebo-treated mice, without affecting the contralateral paw. The analgesic effect of DS peaked within 30 min of injection in a dose-dependent manner, whereas 80 mg/kg gastrodin achieved the optimal effect 1 h after intrathecal injection (Fig. 2b). The analgesic effects of both drugs were stable over four consecutive days post-DS injection (Fig. 2c). In addition, further dose-response analyses showed that DS alleviated allodynia in ipsilateral paw in a dose-dependent manner, with a ED_50_ values of 5.29 mg/kg at 0.5 h after administration DS (Fig. 2d, Figure S1).

### DS inhibited NLRP3 inflammasome activation in the spinal microglia in vivo

**Fig. 3:**
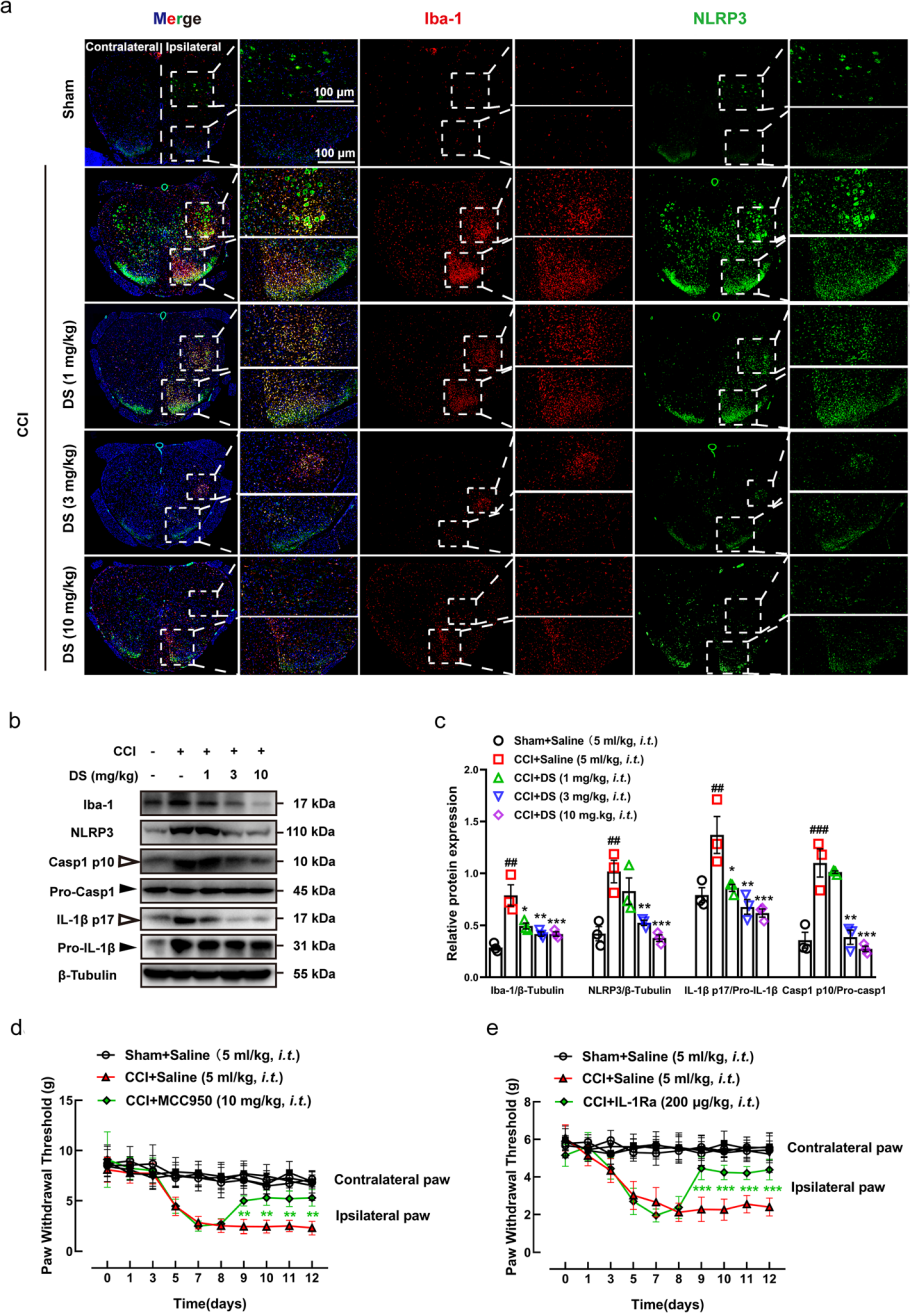
DS showed analgesic effect by inhibiting NLRP3 inflammasome activation of spinal microglia in vivo. **a** The immunofluorescence of the spinal lumbar enlargement of CCI pain mice showed the co-localization of IBA-1 staining (red) and NLRP3 staining (green) in the ipsilateral glial area, and the contralateral side was not affected. **b**, **c** Western blotting analysis of microglia activation marker IBA-1 and NLRP3 inflammasome components in spinal lumbar enlargement tissue showed that DS inhibited the expression of IBA-1, NLRP3 protein, activated caspase-1 p10, and mature IL-1β p17 in a dose-dependent manner. Data are expressed as means ± SEM (*n* = 3 in each group). Statistical significance was determined by one-way ANOVA followed by Tukey’s post hoc analysis where ^##^*P* < 0.01, ^###^*P* < 0.011 vs*.* Sham + Saline group; ^*^P < 0.05, ^**^P < 0.01, ^***^P < 0.001 vs. CCI + Saline group. NLRP3 inflammasome inhibitor MCC950 (**d**) and IL-1R antagonist (**e**) were injected intrathecally into CCI pain mice and the paw withdrawal threshold of their ipsilateral and contralateral paws were measured at 1h post intrathecal injection. Inhibiting the assembly and activation of NLRP3 inflammasome or block the binding of IL-1β to IL-1R can effectively increase the paw withdrawal threshold of CCI mice. Data are expressed as means ± SEM (*n* = 8 in each group). Statistical significance was determined by two-way ANOVA followed by Tukey’s post hoc analysis where ^**^P < 0.01, ^***^P < 0.001 vs. CCI + Saline group

The glial area in the ipsilateral enlarged lumbar spinal cord expressed high levels of the microglial activation marker Iba-1 after sciatic nerve ligation, whereas the contralateral spinal cord was not affected. This clearly indicated spinal microglial activation in the CCI pain model (Fig. 3a). In addition, the activated Iba-1^+^ microglia co-localized with the NLRP3 protein, indicating that neuropathic pain induced by microglial activation is likely related to the activation of NLRP3 inflammasome (Fig. 3a). DS not only inhibited the co-localization of NLRP3 and IBA-1 in spinal microglia but also decreased the in situ expression of IBA-1, NLRP3 protein, activated caspase-1 p10, and mature IL-1β p17 (Fig. 3b, c).

To further explore the role of NLRP3 inflammasome in neuropathic pain, we respectively treated the CCI model mice with MCC950, a specific inhibitor of the NLRP3 inflammasome, and the recombinant IL-1R antagonist (IL-1Ra). As shown in Fig. 3d, e, intrathecal injection of MCC950 and IL-1Ra significantly increased the mechanical withdrawal threshold, and the analgesic effect was stable for four consecutive days after administration. Taken together, IL-1β secreted by the activated NLRP3 inflammasome relays pain signals by binding to the IL-1R expressed on neurons, and the analgesic effect of DS may be related to blocking NLRP3 inflammasome activation and IL-1β/IL-1R signaling in the spinal cord.

### DS inhibited NLRP3 inflammasome activation by promoting autophagic flux formation

**Fig. 4:**
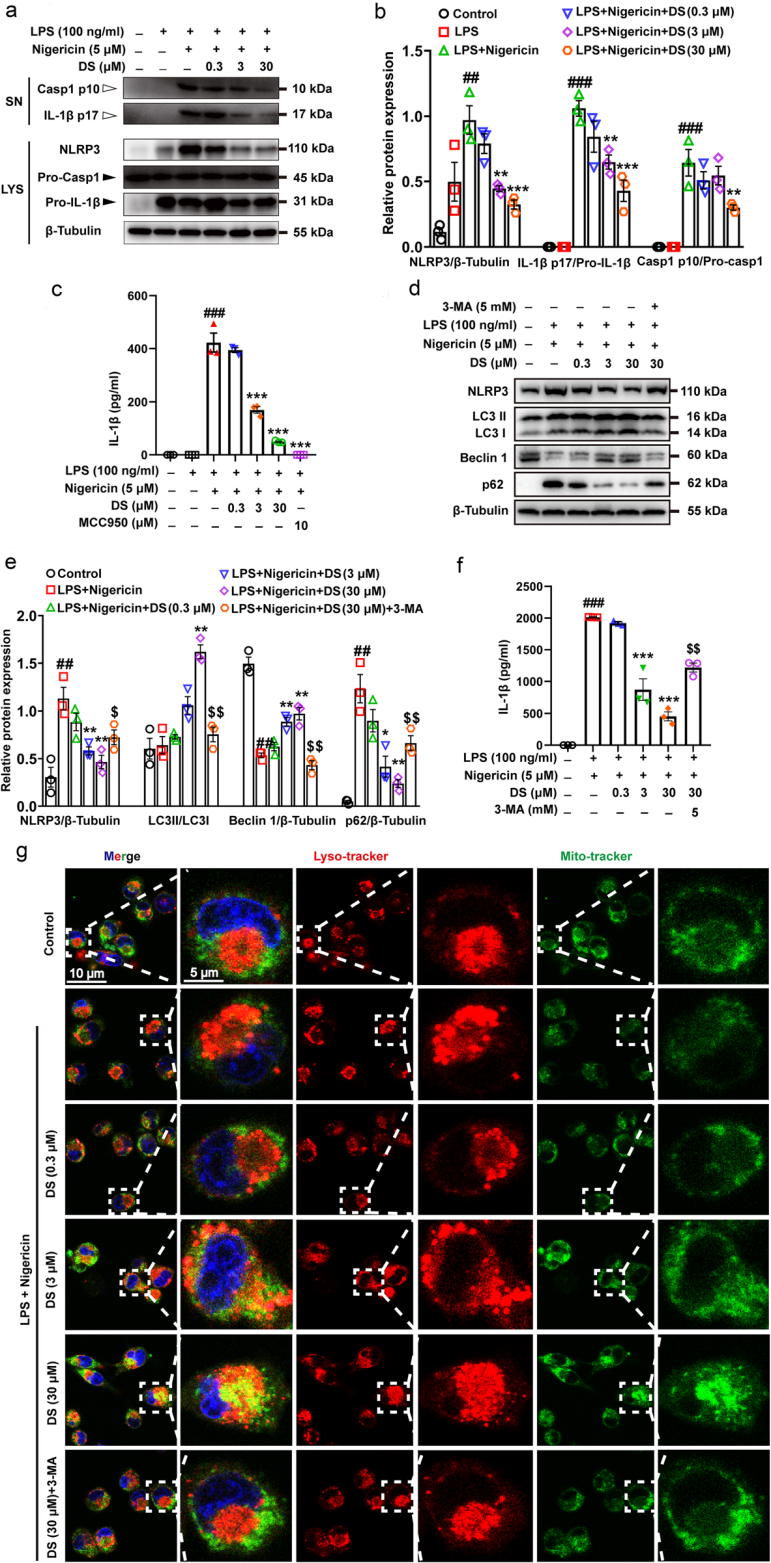
DS inhibited NLRP3 inflammasome activation and promoted mitophagy in microglia in vitro. BV-2 microglia were first pre-stimulated by LPS (100 ng/ml) for 3.5 h, then treated for 1h with indicated concentrations of DS followed by stimulation with nigericin (5 μM) for 30 min. **a**, **b** Immunoblot analyses of caspase-1 p10, IL-1β p17 in culture supernatants (SN) and NLRP3, pro-caspase-1, pro-IL-1β in lysates (LYS) were shown. **c** The level of IL-1β in supernatants was analyzed by ELISA method. After pre-stimulated with LPS, adding autophagy inhibitor 3-MA (5 mM) before DS and incubated for 0.5 h, and finally stimulated with nigericin for 12 h. **d**, **e** Immunoblot analyses of NLRP3, LC3II/I, Beclin 1 and p62 in LYS and **c** ELISA analysis of IL-1β in SN. Data are expressed as means ± SEM (*n* = 3 in each group). Statistical significance was determined by one-way ANOVA followed by Tukey’s post hoc analysis where ^##^*P* < 0.01, ^###^*P* < 0.001 vs. control; ^*^P < 0.05, ^**^P < 0.01, ^***^P < 0.001 vs. LPS + Nigericin group; ^$^*P* < 0.05, ^$$^*P* < 0.01, vs. LPS + Nigericin + 30 μM DS group. **e** Mitochondria and lysosome were respectively labeled with the mito-tracker (green) and lyso-tracker (red), images were captured using Leica TCS SP8 confocal microscope

The in vitro model of NLRP3 inflammasome activation was established by co-stimulating BV-2 microglia with LPS and nigericin. The cells were first exposed to LPS to upregulate the NLRP3 inflammasome components, followed by DS and finally nigericin. DS treatment significantly reduced the expression of NLRP3 in cell lysates (LYS), as well as maturation of caspase-1 and IL-1β in the cultured supernatants (SN), in response to LPS and nigericin (Fig. 4a, b). Similarly, DS also inhibited the nigericin-induced secretion of IL-1β in a dose-dependent manner (Fig. 4c). These findings were similar to that observed in the spinal cord tissues of DS-treated CCI mice. Autophagy flux is a chain process from autophagosome formation to ubiquitin degradation through fusion with lysosomes. Among them, mitophagy play an important role in the phagocytosis of damaged mitochondria. Given the inhibitory role of mitophagy in NLRP3 activation, we next determined whether the therapeutic effects of DS were mediated via mitophagy-induced NLRP3 inflammasome inhibition. Microtubule-associated light chain protein 3 (LC3) is a necessary autophagy marker for the formation of autophagy membrane. In addition, p62 protein is also responsible for the process of autophagy by interacting with LC3 to bring damaged mitochondria and other autophagosomes into autolysosomes for degradation and self-degradation. The results indicated that DS upregulated the expression of LC3II and Beclin 1, increased the ratio of LC3II/LC3I, and decreased the expression of p62 protein and the level of IL-1β in the cultured supernatants (Fig. 4d–f). These data suggested that DS likely promoted mitophagy in the microglia. To better understand the mechanism of increased mitophagy and negative regulation of NLRP3 inflammasome by DS, we also measured the expression of autophagy-associated protein and NLRP3 in the presence of autophagy inhibitor 3-MA. The findings indicated that 3-MA not only blocked the effect of DS on autophagy-related proteins, but also restored NLRP3 activation by increasing the level of IL-1β (Fig. 4d–f). Furthermore, mito-tracker and lyso-tracker were used to trace the formation of autolysosomes during mitophagy in BV-2 microglia. The results indicated that mitochondria damaged by LPS and nigericin were transferred to lysosome for degradation in the process of DS-induced mitophagy, which was also inhibited by the autophagy inhibitor 3-MA (Fig. 4g). These findings suggested that DS promoted the fusion of mitochondria and lysosomes.

**Fig. 5:**
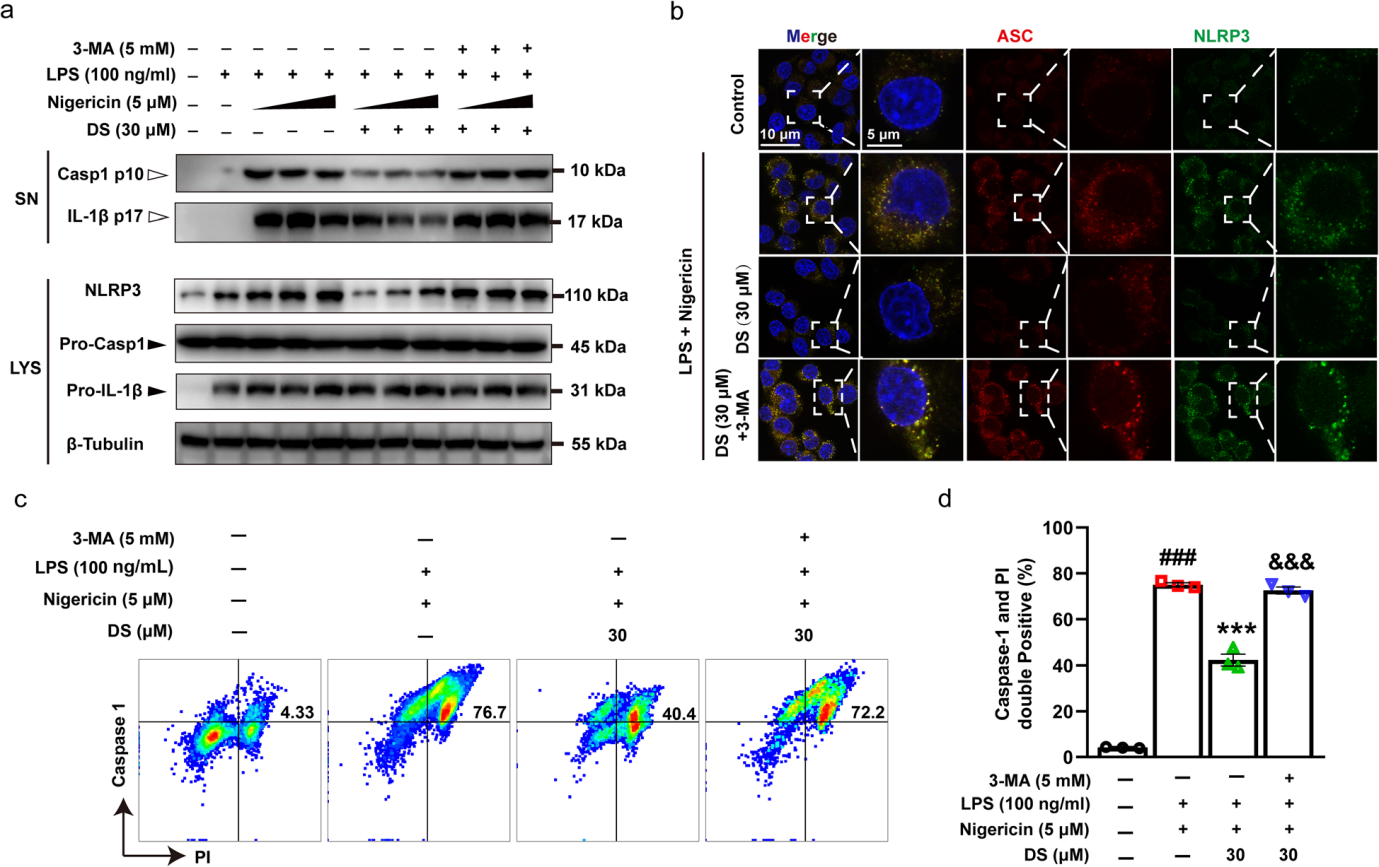
DS negatively regulated the formation of NLRP3 inflammasome complex through mitophagy in BV-2 microglia. BV-2 microglia were first pre-stimulated by LPS (100 ng/ml) for 3.5 h, then treated for 1h with indicated concentrations of DS and 3-MA (5 mM) for 30 min or not followed by stimulation with nigericin (5 μM) for 6 h, 12 h, and 24 h, which was labeled with a triangle at nigericin (5 μM) treatment. **a** Immunoblot analyses of caspase-1 p10, IL-1β p17 in culture supernatants (SN) and NLRP3, pro-caspase-1, pro-IL-1β in lysates (LYS) were shown. **b** Immunofluorescence staining show the expression of NLRP3 (green) and ASC (Red) in BV-2 microglia after stimulation with nigericin for 12 h. **c**, **d** Caspase-1 activity was detected by flow cytometry in BV-2 microglia treated with DS for 12 h using caspase-1/PI double-labeled staining method. Data are expressed as means ± SEM (*n* = 3 in each group). Statistical significance was determined by one-way ANOVA followed by Tukey’s post hoc analysis where ^###^*P* < 0.001 vs. control group; ^***^P < 0.001 vs. LPS + Nigericin group; ^$$$^*P* < 0.001 vs. LPS + Nigericin + 30 μM DS group

To further verify whether DS inhibited the activation of NLRP3 inflammasome through mitophagy, we analyzed the expression levels of individual components of NLRP3 inflammasome in the presence of autophagy inhibitor 3-MA. Our findings showed that DS inhibited the expression of NLRP3 in LYS as well as the secretion of mature caspase-1 p10 and IL-1β p17 in supernatants in the presence of LPS and nigericin, and which could be completely neutralized by 3-MA (Fig. 5a). In addition, our results also indicated that LPS and nigericin induced an obvious overexpression of NLRP3 and ASC in cultured BV-2 microglia, which could be blocked by DS treatment (Fig. 5b). However, 3-MA could reverse the inhibitory effect of DS on the overexpression of NLRP3 and ASC. Furthermore, LPS and nigericin stimulated caspase-1 activation and the conversion from pro-caspase-1 to caspase-1, which appeared as an increase in the population of double-positive cells stained with Caspase-1 and Propidium Iodide (PI) (Fig. 5c, d). Meanwhile, DS obviously inhibited the caspase-1 activity in BV-2 microglia, and this effect could be neutralized by 3-MA (Fig. 5c, d). Taken together, mitochondrial damage induced by LPS and nigericin triggered NLRP3 inflammasome activation. However, DS inhibited formation of NLRP3 inflammasome complex in BV-2 microglia, which was manifested by a decrease in both caspase-1 activity and IL-1β secretion, by inducing mitophagy and promoting clearance of defective mitochondria.

### DS decreased ROS production and inhibited apoptosis in microglia by inducing mitophagy

**Fig. 6:**
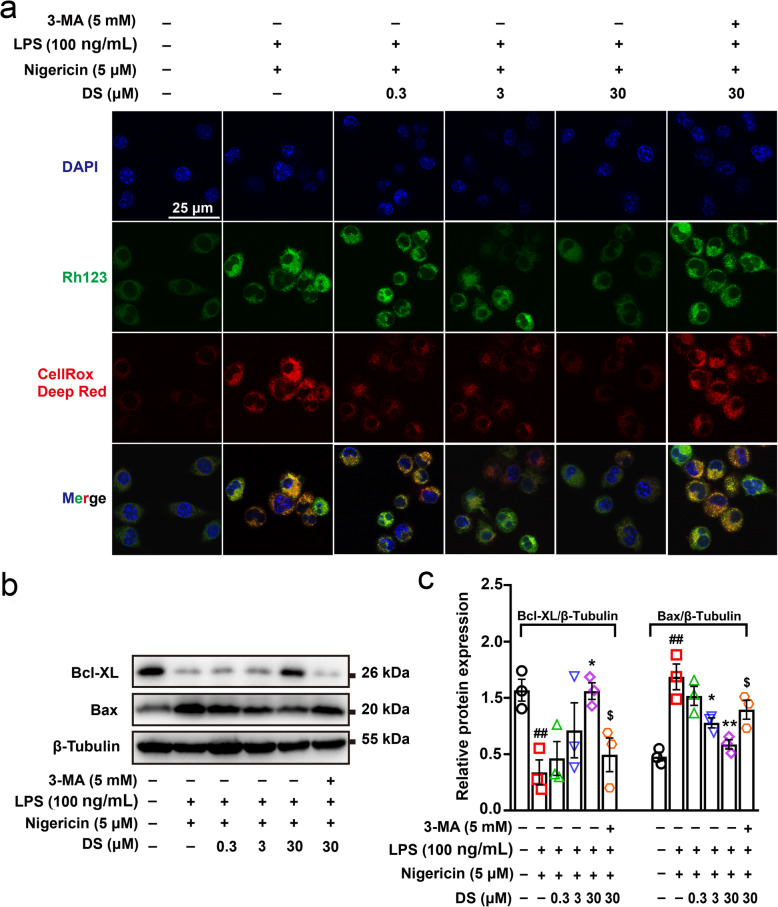
DS promoted autophagic clearance of mitochondrial ROS and protected MMP through mitophagy in microglia. BV-2 microglia were first pre-stimulated by LPS (100 ng/ml) for 3.5 h, then treated for 1 h with indicated concentrations of DS and 3-MA (5 mM) for 30 min or not followed by stimulation with nigericin (5 μM) for 12 h. **a** Immunofluorescence staining Rh123 (green) and CellROX Deep Red (Red) indicate mitochondrial membrane depolarization and ROS production in BV-2 microglia. **b**, **c** Immunoblot analyses the expression of apoptosis associated proteins of Bcl-XL and Bax in lysates. Data are expressed as means ± SEM (*n* = 3 in each group). Statistical significance was determined by one-way ANOVA followed by Tukey’s post hoc analysis where ^##^*P* < 0.01 vs. control group; *P < 0.05, **P < 0.01 vs. LPS + Nigericin group; ^$^*P* < 0.05 vs. LPS + Nigericin + 30 μM DS group

Although our findings indicate that mitophagy induced by DS inhibited the activation of NLRP3 inflammasome in microglia, the underlying mechanism is still unclear. As an important signal to promote the activation of inflammasome, ROS may be involved in the negative regulation of NLRP3 by mitophagy. Therefore, in order to explore whether the analgesic effect of DS was related to autophagic clearance of ROS to inhibit NLRP3 inflammasome activation, the changes of mitochondrial function in microglia with the treatment of DS were assessed. The effect of DS on mitochondrial function was investigated by measuring ROS production and MMP using specific fluorescent probes. As shown in Fig. 6a, NLRP3 inflammasome activation in response to LPS and nigericin significantly increased the fluorescence intensities of both Rh123 and CellROX Deep Red, indicating a high level of mitochondrial membrane depolarization and ROS production respectively. DS dampened the fluorescence intensities of both probes, which was suggestive of MMP restoration and reduction in intracellular ROS levels. Furthermore, 3-MA could counteract the protective effects of DS, indicating that DS promoted ROS clearance in the microglia through mitophagy.

**Fig. 7:**
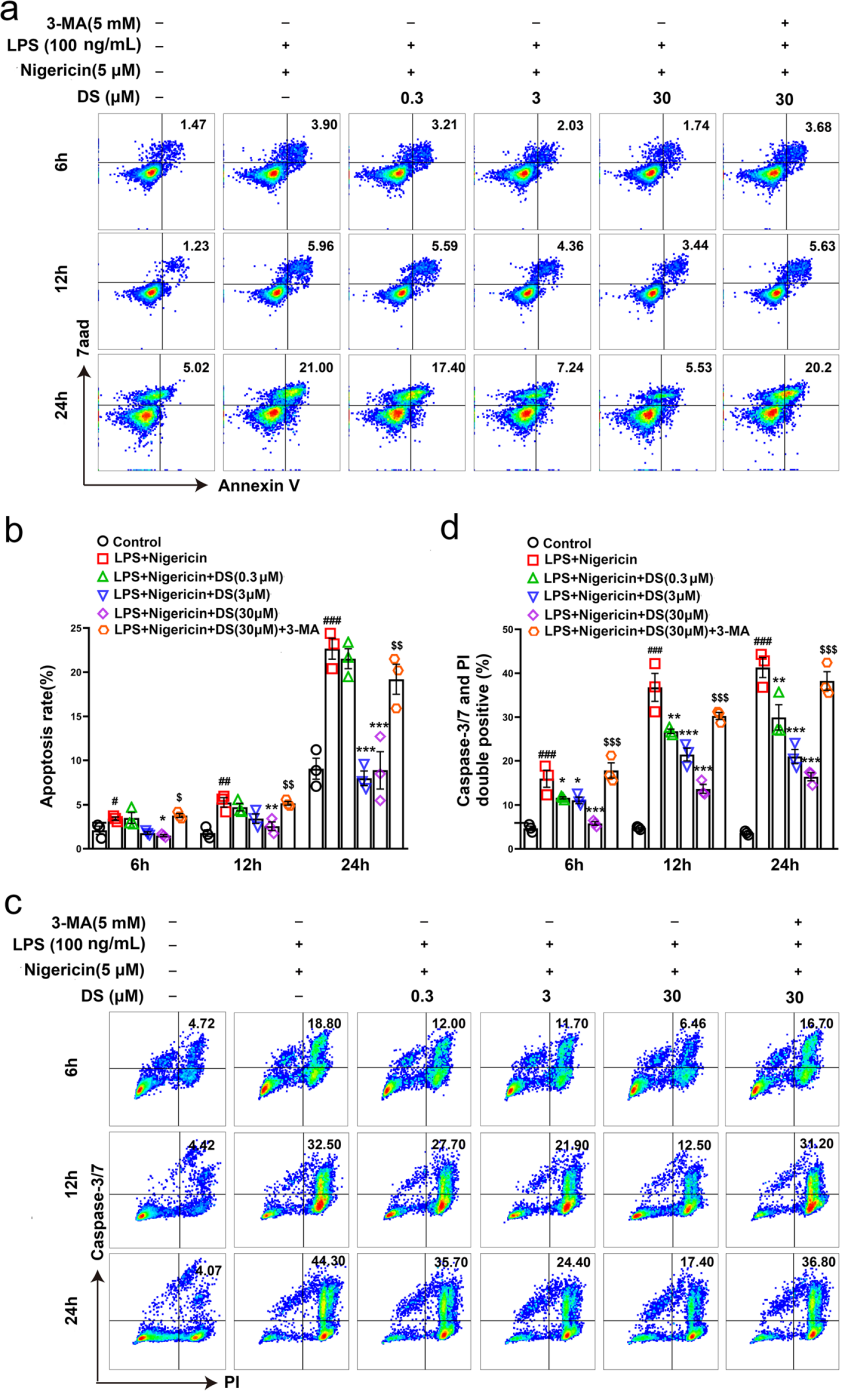
DS inhibited apoptosis and caspase-3/7 activity through mitophagy in BV-2 microglia. BV-2 microglia were first pre-stimulated by LPS (100 ng/ml) for 3.5 h, then treated for 1 h with indicated concentrations of DS and 3-MA (5 mM) for 30 min or not followed by stimulation with nigericin (5 μM) for 12 h. Apoptosis was detected by flow cytometry in BV-2 microglia treated with indicated concentrations of DS for 6, 12, and 24 h using Annexin V-FITC/7aad (**a**, **b**) and caspase-3/7/PI (**c**, **d**) double-labeled staining method. Data are expressed as means ± SEM (*n* = 3 in each group). Statistical significance was determined by one-way ANOVA followed by Tukey’s post hoc analysis where ^#^*P* < 0.05, ^##^*P* < 0.01, ^###^*P* < 0.001 vs. control group; ^*^P < 0.05, ^**^P < 0.01, ^***^P < 0.001 vs. LPS + Nigericin group; ^$^*P* < 0.05, ^$$^*P* < 0.01, ^$$$^*P* < 0.001 vs. LPS + Nigericin + 30 μM DS group

In addition, damaged mitochondria release DAMPs through apoptosis, resulting in a vicious circle of NLRP3 inflammasome activation and mitochondrial dysfunction, and irreversible tissue damage. Therefore, we analyzed the effect of DS on apoptosis to elucidate the potential mechanism underlying NLRP3 inflammasome inactivation. Bax and Bcl-XL are two important proteins involved in the process of apoptosis program. Our research found that DS upregulated the expression of anti-apoptotic Bcl-XL and inhibited the expression of pro-apoptotic Bax in the stimulation of LPS and nigericin which were responsible for formation of NLRP3 inflammasome complex. However, 3-MA could reverse these changes (Fig. 6b, c). Elevated caspase-3/7 activity is an important indicator in the process of apoptosis program. In our study, LPS and nigericin increased the activity of caspase-3/7 and percentage of apoptotic cells in cultured BV-2 microglia. However, DS could block the apoptosis (Fig. 7a, b) and caspase-3/7 activity (Fig. 7c, d) in a time and concentration-dependent manner, which was completely obviated by 3-MA. Taken together, DS promoted mitophagy induced ROS clearance and inhibited apoptosis in activated microglia, which may be responsible for inhibition of NLRP3 inflammasome complex.

### DS ameliorated neuropathic pain through mitophagy-mediated NLRP3 inflammasome inhibition

**Fig. 8:**
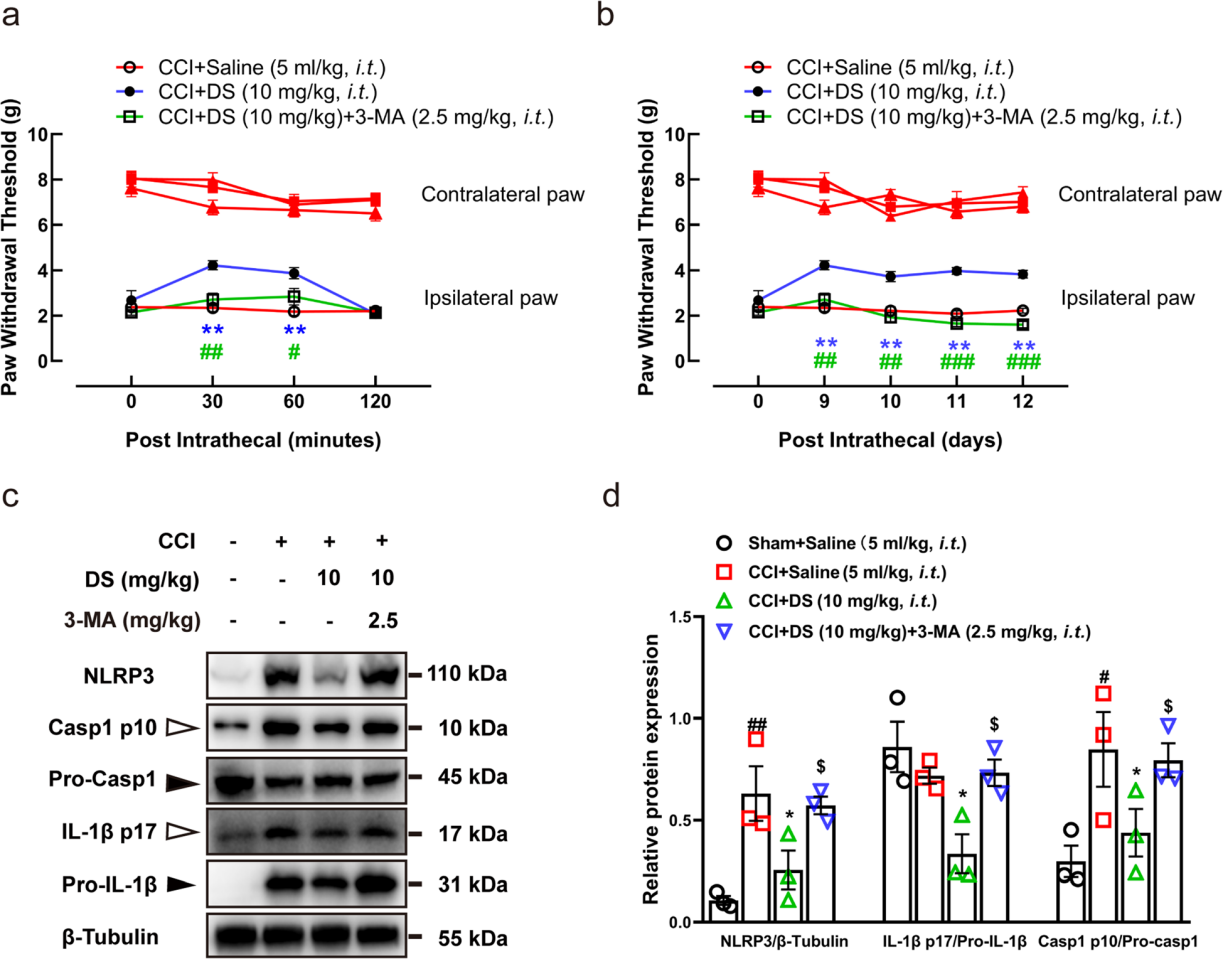
DS ameliorated neuropathic pain through mitophagy-mediated NLRP3 inflammasome inhibition. **a** Paw withdrawal threshold was measured by Von Frey test in both contralateral and ipsilateral paws, after administrating DS (10 mg/kg), DS (10 mg/kg) + 3-MA (2.5 mg/kg), and vehicle (0.9% normal saline) 9 days in CCI-induced model mice, respectively. **b** The analgesic effects of both DS (10 mg/kg) and DS (10 mg/kg) + 3-MA (2.5 mg/kg) treatment over four consecutive days post-injection. Data are expressed as means ± SEM (*n* = 7 mice in each group). Statistical significance was determined by two-way ANOVA followed by Tukey’s post hoc analysis where ^**^P < 0.01 vs. CCI + Saline group, ^#^*P* < 0.05, ^##^*P* < 0.01, ^###^*P* < 0.001 vs. CCI + 10 mg/kg DS group. **c**, **d** Western blotting analysis of NLRP3 inflammasome components in spinal lumbar enlargement tissue. Data are expressed as means ± SEM (*n* = 3 in each group). Statistical significance was determined by one-way ANOVA followed by Tukey’s post hoc analysis where ^#^*P* < 0.05, ^##^*P* < 0.01 vs*.* Sham + Saline group; ^*^P < 0.05 vs. CCI + Saline group; ^$^*P* < 0.05 vs. CCI + 10 mg/kg DS group

To further explore the possible mechanism DS exerting analgesic effect, autophagy inhibitor 3-MA was applied in our research trying to clarify the regulation of mitophagy on NLRP3 inflammasome. On day nine of the CCI pain model, 3-MA was administrated by intrathecal injection 30 min before the administration DS, and the bilateral paw withdrawal threshold of each group of CCI mice were measured at 30 min, 1 h, and 2 h after administration DS. The results showed that DS could obviously increase the pain threshold of CCI mice and reach the peak of analgesia 30 min after administration DS. However, the autophagy inhibitor 3-MA could block the analgesic effect of DS (Fig. 8a). After four consecutive days of same administration, it was found that the analgesic effect of DS was relatively stable and no obvious tolerance occurred (Fig. 8b). In addition, four consecutive days of 3-MA treatment not only reversed the effect of DS, it even lowered the pain threshold than that of the model group (Fig. 8b), which suggested that mitophagy played an important role in the occurrence and development of neuralgia, and DS might exert an analgesic effect by inducing mitophagy.

Further analysis by Western blot found that the inhibitory effect of DS on the activation of NLRP3 inflammasome in the spinal cord tissue of CCI mice was countered by 3-MA (Fig. 8c, d), indicating that mitophagy played a negative regulatory role on the activation of NLRP3 inflammasome in CCI-induced pain model. These data indicated that DS may inhibit the activation of NLRP3 inflammasome signaling via inducing mitophagy in CCI-induced neuropathic mice, thereby reducing the pain signal conduction caused by the binding of IL-1β to IL-1R on the spinal dorsal horn neurons, therefore raising the pain threshold.

## Discussion

Microglia account for 5–10% of all cells in the CNS and play an important role in innate immune responses. However, aberrantly activated microglia can trigger neuroinflammation and tissue damage [18, 37]. Peripheral and central nerve damage in animal models of neuropathic pain are accompanied by the upregulation of inflammatory factors, chemokines, brain-derived neurotrophic factor (BDNF), toll-like receptors (TLRs), and purinergic receptor in the microglia [38, 39]. Thus, aberrant phenotypic and functional changes in microglia accompany the initiation and development of neuropathic pain, which involve NLRP3 inflammasome activation [14, 40]. PNI induces a release of PAMPs and DAMPs in glial region of the spinal cord, which are recognized by the microglia. PAMPs activate the priming signal of NLRP3 inflammasome, and DAMPs act as the second signal that promote neuroinflammation and facilitate transmission of pain signals from the primary center to the superior center [11, 14, 20, 21]. Blocking NLRP3 inflammasome activation in spinal cord microglia could suppress PNI-induced pain hypersensitivity [41, 42]. Mitophagy is a conserved defense mechanism in eukaryotic cells, and has been implicated in the inhibition of NLRP3 inflammasome activation and neuropathic pain [29, 43, 44]. However, the exact mechanistic basis is not completely known. In this study, we assessed the analgesic properties of DS, derivative from an active constitute of the traditional Chinese medicine Gastrodia, and established the functional link between mitophagy-mediated inhibition of NLRP3 inflammasome activation and alleviation of neuropathic pain.

Microglia acts as a kind of typical brain-resident macrophages to phagocytose the damaged debris in CNS. During the chronic neuropathic pain injury, microglia is activated and polarized into pro-inflammatory phenotype and chronic neuroinflammation [45]. In the CCI pain model as well, sciatic nerve ligation increased IBA-1-positive microglia in the spinal lumbar area; this is accompanied by a gradual decrease in the mechanical pain threshold on the ligated side. It has been reported that activated microglia co-localized with NLRP3 protein, indicating NLRP3 inflammasome activation in the microglia [10, 25]. The central sensitization and pain development caused by microglial activation are related to the activation of NLRP3 inflammasome. For example, the NLRP3 inflammasome inhibitor MCC950 could increase the pain threshold of CCI mice. In addition, the NLRP3 inflammasome effector IL-1β is an important pro-inflammatory factor that promotes pain signal transduction [18, 26, 46]. IL-1Ra inhibited the IL-1β/IL-1R signaling and increased the pain threshold of CCI mice, further underscoring the role of NLRP3 inflammasome in neuralgia. It was found that DS significantly alleviated the neuropathic pain by increasing mechanical withdrawal threshold in CCI-induced neuropathic pain mice. Meanwhile, DS effectively inhibited the activation of NLRP3 inflammasome in LPS and nigericin-stimulated BV-2 microglia cells by suppressing caspase-1 activation and IL-1β secretion. Given that DS affects the expression of NLRP3 inflammasome-associated proteins in activated BV-2 cells and CCI-induced neuropathic pain model mice, as well as DS or NLRP3 inflammasome inhibitor MCC950 and IL-1Ra all can ameliorate the neuropathic pain by increasing the mechanical withdrawal threshold, it is suggested that DS reduce the nociception likely via NLRP3 inflammasome mechanism. Thus, our work hints that focus on inhibiting NLRP3 inflammasome activation might be a new therapeutic strategy for chronic neuropathic pain.

Increasing evidence suggests that damaged mitochondria involved in the progress of disease with stress, ROS production, and NLRP3 inflammasome activation, including chronic neuropathic pain. An important role of mitophagy is to selectively remove excess or damaged mitochondria. Following ROS-induced depolarization, the damaged mitochondria are engulfed by the autophagosomes that fuse with the lysosomes, eventually reducing intracellular ROS levels and inhibiting NLRP3 inflammasome activation [47, 48]. During mitophagy, the endoplasmic reticulum membrane wraps the damaged mitochondria to form autophagosomes, and the subsequent modification of the LC3-I protein to LC3-II protein promotes their fusion with lysosomes [49, 50]. The autophagic flux is also driven by degradation of the ubiquitin-binding protein p62 [51], and the dissociation of Beclin-1 from the anti-apoptotic Bcl-2 or Bcl-XL [52]. Beclin-1 cleavage by caspases on the other hand inhibits autophagy and triggers the apoptotic cascade by promoting cytochrome C release from mitochondria [53, 54]. In this study, we found that DS alleviated the mitochondrial damage and ROS generation induced by mitochondrial membrane depolarization in LPS and nigericin-stimulated microglia by promoting mitophagy. Therefore, our research further confirmed that mitophagy contributed to the process of mitochondrial protection and ROS clearance in excessive activated microglia.

Mitophagy blockade is often accompanied by severe mitochondrial damage and ROS production, and the subsequent activation of NLRP3 inflammasome [55, 56]. Therefore, the autophagic flux is a potential therapeutic target in NLRP3 inflammasome-related diseases. Chronic neuropathic pain is characterized by mitochondrial dysfunction and accumulation of ROS in the glial area of the spinal cord, which act as DAMPs for the NLRP3 inflammasome activation. Notably, autophagy increases mechanical pain threshold in neuropathic pain models, likely by inhibiting NLRP3 inflammasome activation [40, 57]. However, the mechanism underlying the regulatory role of mitophagy-mediated NLRP3 inflammasome on chronic neuropathic pain remain poorly understood. Here, our research indicated that DS induced an obvious mitophagy and ROS reduction in cultured microglia. To determine the role of mitophagy on NLRP3 inflammasome activation, the mitophagy/autophagy inhibitor 3-MA was used to investigate the regulation for NLRP3 inflammasome under DS treatment. The results showed that 3-MA neutralized DS-induced mitophagy in the activated microglial cells, which hindered degradation of damaged mitochondria and removal of ROS. Moreover, DS-mediated inhibition of NLRP3 inflammasome activation and mature IL-1β secretion from the microglia were also blocked by 3-MA. In vivo experiment further also verified that 3-MA could prevent the analgesic effect of DS in CCI-induced neuropathic pain model mice. Therefore, these data indicated that DS-induced mitophagy is accompanied by inactivation of NLRP3 inflammasome. Briefly, the current findings suggested that the effect of DS on chronic neuropathic pain may be associated with mitophagy induction, which mediates the NLRP3 inflammasome inactivation and neuroinflammatory inhibition.

## Conclusion

**Fig. 9:**
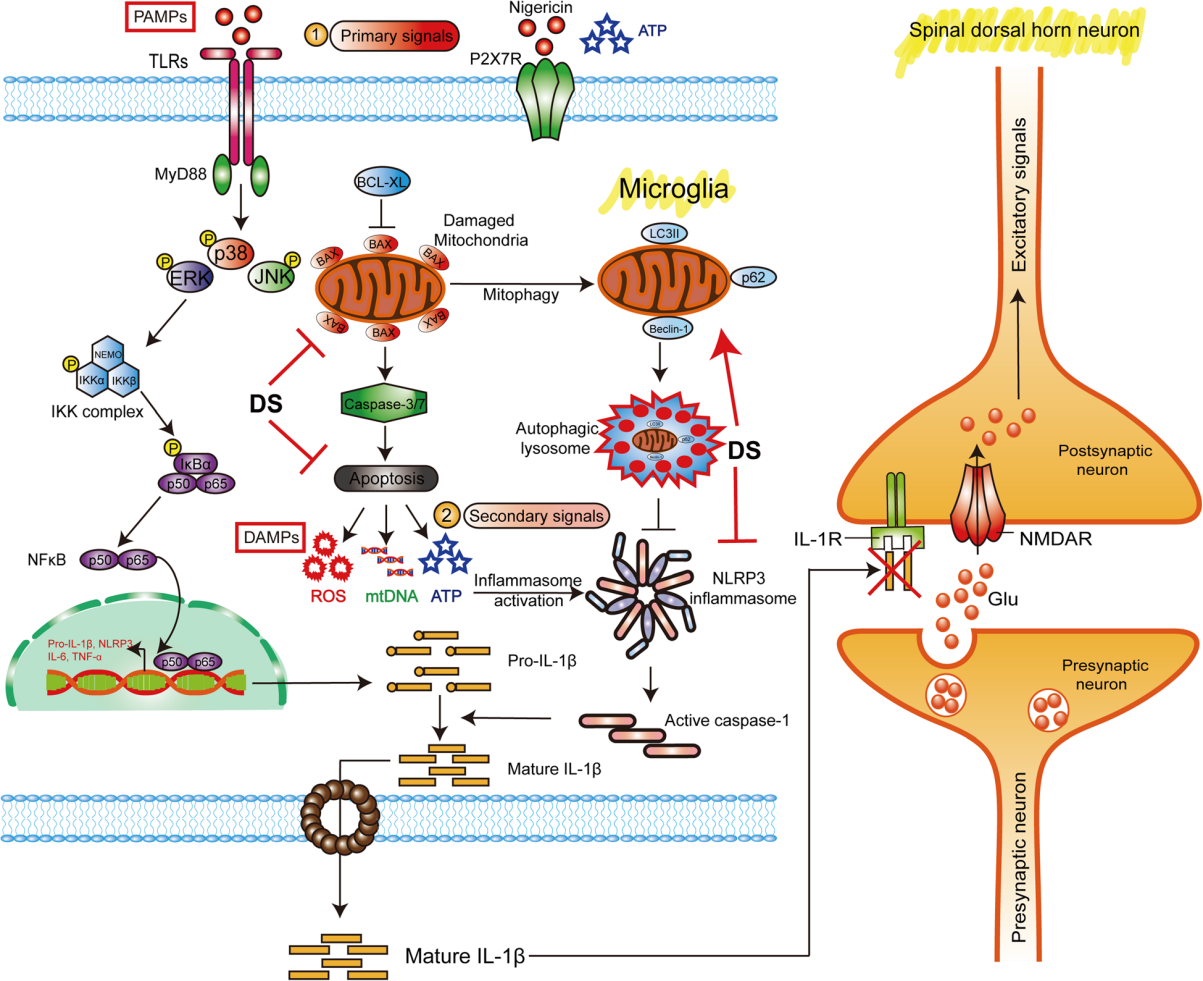
Proposed analgesic mechanism of DS for mitophagy-induced NLRP3 inflammasome inhibition in spinal microglia

In summary, we reported that DS effectively improved the chronic neuropathic pain in CCI-induced neuropathic pain model mice. The underlying mechanisms have been investigated that DS promoted rapid clearance of ROS released by dysfunctional mitochondria through mitophagy, and further suppressed the NLRP3 inflammasome activation and neuroinflammation (Fig. 9). Our work has revealed the negative regulation of mitophagy to NLRP3 inflammasome in neuropathic pain, which suggested that NLRP3 inflammasome may be a promising therapeutic strategy for chronic neuropathic pain. The findings also provide a potential candidate for treatment of chronic neuropathic pain.

## Supplementary Information

**Additional file 1 .**

## Data Availability

The datasets during and/or analyzed during the current study are available from the corresponding author on reasonable request.

## Abbreviations

DS: Divanillyl sulfone
IL-1β: Interleukin-1β
IL-18: Interleukin-18
IL-1R: Interleukin-1 receptor
IL-1Ra: Interleukin-1 receptor antagonist
NLRP3: NLR family pyrin domain-containing 3
CNS: Central nervous system
DRG: Dorsal root ganglia
PRRs: Pattern recognition receptors
PAMPs: Pathogen-associated molecular patterns
DAMPs: Danger-associated molecular patterns
SDH: Spinal dorsal horn
NMDAR: *N*-methyl-d-aspartate receptor
GABA: γ-Aminobutyrate
LPS: Lipopolysaccharide
3-MA: 3-Methyladenine
CCI: Chronic constrictive injury
SN: Supernatants
LYS: Lysates
PBS: Phosphate buffer saline
MMP: Mitochondrial membrane potential
ROS: Reactive oxygen species
LC3: Microtubule-associated light chain protein 3
p62: Protein 62
