# Supplementary material for: Divanillyl sulfone suppresses NLRP3 inflammasome activation via inducing mitophagy to ameliorate chronic neuropathic pain in mice
Source: J Neuroinflammation. 2021 Jun 24;18:142. doi: 10.1186/s12974-021-02178-z (PMC8223331; doi:10.1186/s12974-021-02178-z)
Supplement: Supplementary file 1 — Additional file 1 . [file 12974_2021_2178_MOESM1_ESM.docx]

**Supplemental Information**

**Divanillyl sulfone suppresses NLRP3 inflammasome activation via inducing mitophagy to** **ameliorate chronic neuropathic pain in mice**

Shuai Shao^†^, Cheng-Bo Xu^†^, Cheng-Juan Chen, Gao-Na Shi, Qing-Lan Guo, Yu Zhou, Ya-Zi Wei, Lei Wu, Jian-Gong Shi^*^ and Tian-Tai Zhang^*^

State Key Laboratory of Bioactive Substances and Functions of Natural Medicines, Institute of Materia Medica, Chinese Academy of Medical Sciences & Peking Union Medical College, Beijing 100050, China

^*^ Correspondence: ttzhang@imm.ac.cn; shijg@imm.ac.cn.

^†^ Shuai Shao and Cheng-bo Xu contributed equally to this work.

**Supplemental Inventory**

Figure S1, Related to Figure 1

**Author contributions**

**
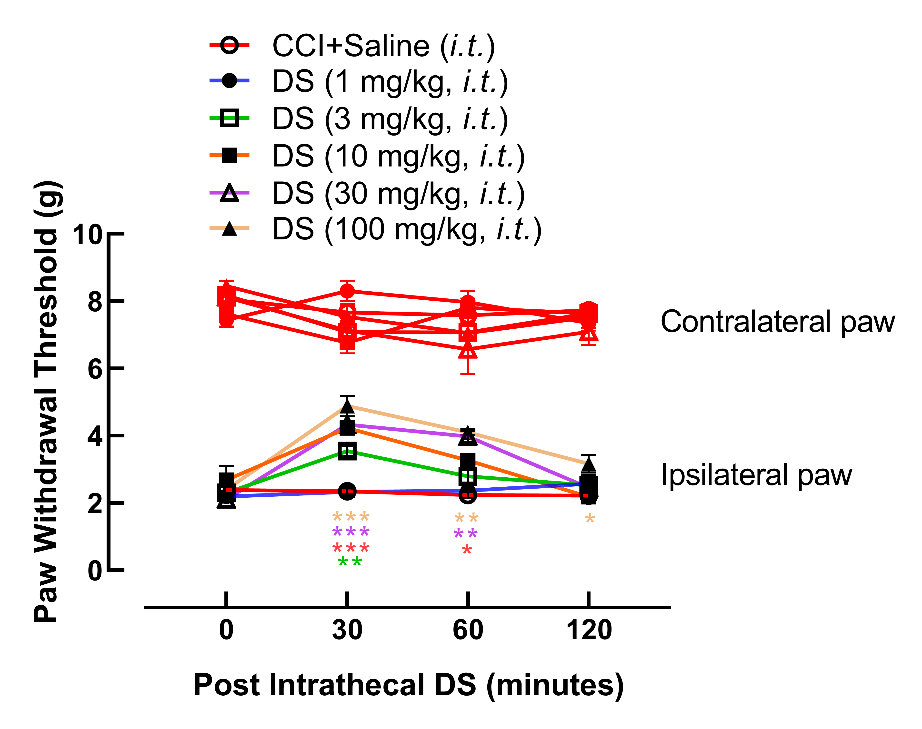
**

**Figure S1. Analgesic effect of DS administered intrathecally in neuropathic pain mice, induced by one-sided sciatic nerve ligation.** Neuropathic pain mice received intrathecal injection of DS (1, 3, 10, 30 and 100 mg/kg) and the paw withdrawal threshold at 30, 60, 120 minutes after injection were measured by Von Frey test in both contralateral and ipsilateral paws. Data are expressed as means ± SEM (n = 7 mice in each group). Statistical significance was determined by two-way ANOVA followed by Tukey’s post hoc analysis where ^*^*P* < 0.05, ^**^*P* < 0.01, ^***^*P* < 0.001 vs. CCI + Saline group.

**Author contributions**

SS and TTZ designed the research study and wrote the paper. XCB, GQL and SJG isolated and synthesize the compound. SS, CCJ, SGN and ZY performed the experiment. WYZ and WL analyzed the data.
